# High-throughput chemical screen identifies epothilone B in modulating inflammatory bowel disease by triggering neutrophil apoptosis

**DOI:** 10.1016/j.gendis.2025.102009

**Published:** 2025-12-23

**Authors:** Zhenting He, Ziling Deng, Huan Zhang, Yiming Xiang, Jiashi Guo, Yi Chen, Jingjing Qin, Ling Meng, Fengmin Xu, Xiaowen Wang, Cheng Zhang, Siyuan Hou, Chengcheng Tao, Ruijie Li, Jun Chen, Yue Wei, Wei Wang, Hao Yuan, Chunguang Ren

**Affiliations:** a Laboratory of Developmental Biology, School of Basic Medical Sciences, Chongqing Medical University, Chongqing 400042, China; b Shanghai Institute of Hematology, Sino-French Research Center for Life Sciences and Genomics, State Key Laboratory of Medical Genomics, National Research Center for Translational Medicine at Shanghai, Ruijin Hospital, Shanghai Jiao Tong University School of Medicine, Shanghai 200025, China; c Department of Clinical Laboratory, Nanchong Central Hospital (Nanchong Hospital of Beijing Anzhen Hospital, Capital Medical University), The Second Clinical Medical College of North Sichuan Medical College, Nanchong, Sichuan 637000, China; d Department of Cardiothoracic Surgery, The First Affiliated Hospital of Chongqing Medical University, Chongqing Medical University, Chongqing 400042, China; e Chongqing Depu Foreign Language School, Chongqing 401320, China; f College of Pharmacy, Chongqing Medical University, Chongqing 400016, China; g Basic Medicine Research and Innovation Center for Novel Target and Therapeutic Intervention (Ministry of Education), College of Pharmacy, Chongqing Medical University, Chongqing 400016, China; h Department of Immunology and Microbiology, Zhongshan School of Medicine, Sun Yat-sen University, Guangzhou, Guangdong 510080, China

**Keywords:** Apoptosis, Epothilone B, High-throughput chemical screen, Inflammatory bowel disease, Neutrophil

## Abstract

Inflammatory bowel disease (IBD) is a chronic inflammatory condition of the gastrointestinal tract characterized by a complex interplay of genetic, environmental, and immunological factors. Neutrophils, as a key component of the innate immune system, play a critical role in IBD pathogenesis due to their dysregulated infiltration, impaired apoptosis, and resultant epithelial damage during the disease process. Conventional approaches aimed at suppressing neutrophil activity have achieved only modest clinical efficacy and highlight the need for strategies that recalibrate neutrophil responses without compromising their essential antimicrobial functions. In this study, we employed high-throughput chemical screening using neutrophil-specific transgenic zebrafish larvae to identify compounds capable of modulating neutrophil homeostasis, and subsequently evaluating their efficacy in a dextran sodium sulfate (DSS)-induced IBD model. We identified epothilone B (Epo B), a traditional chemotherapeutic drug, as a potential therapeutic candidate for IBD. Our findings demonstrate that Epo B protects against IBD by promoting intestinal epithelial recovery, alleviating inflammatory responses, and rebalancing the gut microbiota. Mechanistically, Epo B selectively stimulates neutrophil apoptosis by targeting and enhancing Caspase-3 cleavage, thereby inhibiting neutrophilic inflammation. This study underscores the utility of *in vivo* high-throughput drug screening in IBD and highlights Epo B as a promising candidate for drug repurposing in IBD treatment, offering a novel therapeutic strategy by targeting neutrophil apoptosis.

## Introduction

The etiology of inflammatory bowel disease (IBD), which encompasses Crohn’s disease and ulcerative colitis, remains complex and involves genetic predispositions, alterations in microbiota, and immune dysregulation.^1^ Although biological therapies, such as inhibitors of tumor necrosis factor and Janus kinase, are increasingly utilized in the treatment of patients with IBD, the disease remains relapsing and life-threatening, with rising incidence and prevalence worldwide. Novel therapeutic strategies are being explored to address these challenges.

Neutrophils are increasingly recognized to play a dual role in IBD, exerting both protective and deleterious effects on intestinal homeostasis.^2^^,^^3^ During active disease, massive recruitment and hyperactivation of neutrophils lead to the release of reactive oxygen species (ROS), cytotoxic proteases, neutrophil extracellular traps (NETs), and pro-inflammatory cytokines, thereby amplifying inflammation and aggravating mucosal injury. However, neutrophils are also indispensable for host defense, because they eliminate invading microorganisms and generate pro-resolving mediators that support epithelial barrier maintenance and tissue repair. Under physiological conditions, neutrophils undergo rapid apoptosis and subsequent clearance by macrophages, but inflammatory cues in IBD delay neutrophil apoptosis, prolonging their lifespan and promoting their accumulation within the mucosa. This sustained survival of neutrophils enhances epithelial barrier disruption through the continued release of ROS and cytotoxic granules such as myeloperoxidase and NETs.^3^, ^4^, ^5^ Despite their pathogenic contribution, current neutrophil-targeting strategies, such as antibody-mediated depletion or inhibition of neutrophil trafficking, have yielded limited therapeutic benefit in IBD, as they reduce inflammation but frequently impair host defense and increase infection susceptibility.^2^ These therapeutic limitations underscore the need for strategies that fine-tune, rather than abolish, neutrophil activity. Compared to traditional approaches, systematic chemical screening enables the identification of small molecules that selectively regulate neutrophil survival or functional states without completely abolishing neutrophil activity.^6^^,^^7^ This approach provides an opportunity to discover compounds that fine-tune neutrophil responses to reduce tissue damage while preserving essential protective functions.

The zebrafish genome exhibits a high degree of homology with the human genome, particularly with respect to its innate immune system. Approximately 82% of genes associated with human diseases have corresponding orthologs in zebrafish.^8^ Zebrafish embryos and larvae are exceptionally useful *in vivo* models for high-throughput chemical screening of drugs, because of their small size, optical transparency, rapid reproduction, and ease of drug exposure in fish culture media.^9^^,^^10^ IBD-like conditions have been successfully established using chemicals such as dextran sodium sulfate (DSS) and 2,4,6-trinitrobenzenesulfonic acid (TNBS). These zebrafish or mouse models closely emulate various characteristics of human IBD, particularly the similarities in compromised intestinal barrier function, elevated pro-inflammatory cytokine levels, inflammatory cell infiltration, oxidative stress, and histopathological features.^6^^,^^11^, ^12^, ^13^

In this study, we conducted a high-throughput screening of 1430 Food and Drug Administration (FDA)-approved drugs and identified several compounds that alter neutrophil homeostasis in zebrafish, resulting in either an increase or decrease in neutrophil counts. Subsequently, we evaluated the effects of these identified compounds in a DSS-induced zebrafish enterocolitis model and observed that epothilone B (Epo B) exerted a protective effect against IBD by enhancing intestinal epithelial recovery, suppressing inflammatory responses, and restoring the gut microbiota in zebrafish. Furthermore, we demonstrated that Epo B selectively induced neutrophil apoptosis without affecting differentiation, leading to reduced neutrophilic inflammation and alleviation of gut injury in zebrafish and mouse models of IBD. Our study suggests that Epo B could serve as a novel therapeutic agent for IBD by targeting neutrophil apoptosis.

## Materials and methods

### Zebrafish and mice husbandry

The wild-type (WT) AB strain and transgenic zebrafish lines, including *Tg*(*mpx*:*GFP*)^*i114*^,^14^ *Tg*(*mpeg1*:*Gal4*)^*gl24*^;*Tg*(*UAS*:*Nfsb-mCherry*)^*i1*49^,^15^ *Tg*(*gata1*:*DsRed*),^16^ *Tg*(*kdrl*:*mCherry*)^*is5*^^17^ and *Tg*(*cd41*:*GFP*),^18^ were raised and maintained under standard conditions as previously described.^7^ Female C57BL/6 mice (8-week-old) were obtained from the Vital River Laboratories (Beijing, China). All mice were maintained under specific pathogen-free conditions in individually ventilated cages on a 12-h light/dark cycle, with *ad libitum* access to standard chow and water. All animal studies were approved by the Institutional Animal Care and Use Committee of Chongqing Medical University (Approval No. IACUG-CQMU-2024-0139).

### High-throughput screening and high-content image assay

Zebrafish embryos collected at 30 h post-fertilization (hpf) were treated with 2 mg/mL pronase (Roche) for 5 min to remove embryonic chorions. The embryos were subsequently placed into a Cell Carrier-96 well plate, with five embryos per well, containing 200 μL of E3 culture medium. A total of 1430 compounds from an FDA-approved drug library (L1300, Selleck Chemicals, Houston, TX, USA) were dissolved in dimethyl sulfoxide (DMSO, Solarbio, Beijing, China) and added to wells of a 96-well plate at a final concentration of 10 μM using a JANUS liquid-handling workstation (PerkinElmer, Waltham, MA, USA). The plate was agitated for 1 min on a microplate shaker to ensure thorough mixing. The embryos were incubated in a culture chamber at 28.5 °C until 72 hpf. Prior to imaging, 2 μL of 0.4% Tricaine (A5040, Sigma–Aldrich, St. Louis, MO, USA) was added to each well for anesthetic purposes. The Opera Phenix high-content imaging system (PerkinElmer) was employed to scan and capture images, with one field of view per well. The acquired images were analyzed using Harmony 4.1 software to quantify the cell number and fluorescence intensity of green fluorescent protein (GFP)-positive neutrophils within each well.

### Live imaging of zebrafish

Following tricaine treatment, embryos and larvae were mounted on imaging plates using 4% methylcellulose. During imaging, embryos and larvae were maintained at 28.5 °C. Fluorescence and bright-field images were captured using a Zeiss SteREO Discovery (V12) fluorescence stereoscope. The imaging was performed in a blinded manner.

### Induction of IBD animal models

The DSS-induced zebrafish enterocolitis protocol was adapted and modified from a published method.^19^ Briefly, to induce colitis, embryos at 3 days post-fertilization (dpf) were incubated in freshly prepared 0.5% (w/v) DSS (molecular weight 36,000–50,000, 160110; MP Biomedical, Santa Ana, CA, USA) for 3 days. Dexamethasone (DEX) and Z-VAD-FMK (zVAD) were purchased from TargetMol (Boston, USA). All drugs were dissolved in DMSO and administered to the larvae for 24 h (5–6 dpf). The working concentrations of the hit compounds were listed in Table S1. The working concentrations of DEX and zVAD are 100 and 300 μM, respectively.

For the induction of murine acute colitis, 8-week-old female mice were randomly assigned to control and Epo B treatment groups. All mice received drinking water containing 2% (w/v) DSS *ad libitum* for 5 consecutive days, followed by regular drinking water for 3 days. Mice in the Epo B treatment group were administered intraperitoneal injections of Epo B (0.1 mg/kg) on days 1, 3, and 5 during DSS administration, while control mice received vehicle injections under the same schedule. Body weight, stool consistency, and fecal occult blood were monitored daily to assess the disease activity index (DAI). The colon was excised from the cecum to the rectal end following euthanasia and laid flat on a sterile surface. The total colon length was then measured immediately using a precision ruler.

### Histological analysis

Zebrafish larvae at 6 dpf and mouse colonic tissue rings were fixed in 4% paraformaldehyde (PFA) in PBS overnight at 4 °C, paraffin-embedded, and sectioned at 5 μm for hematoxylin and eosin (H&E) staining. Histological evaluation was performed according to species-specific scoring systems adapted from previously described methods.^20^^,^^21^ For zebrafish, three intestinal regions—the intestinal bulb (IB), mid-intestine (MI), and posterior intestine (PI)—were assessed and integrated into a composite score per larva. Grading criteria included epithelial integrity, villus deletion, gut lumen expansion, vacuolation, and goblet cell proportion (each 0–3). At least five larvae were analyzed per condition. For mice, colonic inflammation was scored by two blinded pathologists using a four-point scale (0–3). Scores reflected the extent of inflammatory cell infiltration and mucosal architectural changes, including crypt distortion, enhanced proliferation, ulceration, or erosion. For each colon, 2–6 sections were evaluated.^22^

### Resazurin reduction assay

The assay was performed as previously described with minor modifications.^19^ Briefly, larvae collected at 6 dpf were incubated in a solution of 1 mg/mL Alamar blue (R7017, Sigma–Aldrich) for 30 min. The larvae were anesthetized and individually imaged using a fluorescent microscope. The fluorescence intensity was quantified using Image J software version 1.43 (National Institutes of Health, Bethesda, MD, USA).

### Alcian blue staining

To visualize and quantify the intestinal goblet cells, zebrafish larvae were subjected to Alcian blue staining. Briefly, 6 dpf-larvae were anesthetized and fixed overnight at 4 °C in 4% PFA. The sectioned samples were washed with PBS, dehydrated through a graded ethanol series, and rehydrated. The specimens were then incubated in 0.001% Alcian blue solution (in 20% acetic acid, pH 2.5) for 15 min at room temperature. Excess stain was removed by rinsing with 3% acetic acid, followed by three washes in PBS. The stained sections were mounted on glass slides and examined under a microscope to calculate the number of Alcian blue-positive cells in the posterior intestine.

### *In situ* detection of apoptotic cells and neutrophils

Apoptotic cells and neutrophils were detected using the terminal deoxynucleotidyl transferase dUTP nick-end labeling (TUNEL) assay. For whole-mount TUNEL assay in zebrafish, *mpx*:*GFP* larvae collected at 6 dpf were anesthetized and fixed in 4% PFA in PBS overnight at 4 °C. After washing with 0.1% Tween-20 in PBS, the larvae were permeabilized with proteinase K (10 mg/mL) and refixed with 4% PFA. Apoptotic cells were labeled using the zebrafish TUNEL (Cy3) staining kit (Hunter Biotechnology, Hangzhou, China) following the manufacturer’s instructions. Fluorescence images were captured with a confocal laser scanning microscope (AX/AX R, Nikon, Tokyo, Japan). The number of apoptotic neutrophils was determined by counting TUNEL and GFP double-positive cells within the caudal hematopoietic tissue (CHT) using ImageJ software.

Apoptosis of neutrophils and macrophages in mouse colon tissues was assessed by co-staining TUNEL assay with cell-specific markers (Ly6G for neutrophils, F4/80 for macrophages). Briefly, paraffin-embedded sections were subjected to antigen retrieval, blocking, and incubation with primary antibodies followed by fluorophore-conjugated secondary antibodies. Nuclei were visualized with DAPI. Fluorescent images were acquired with fluorescence microscopy under consistent settings. The percentage of Ly6G^+^/TUNEL^+^ or F4/80^+^/TUNEL^+^ double-positive cells relative to total marker-positive cells was quantified in a blinded manner from at least three sections per mouse, with three to five high-power fields per section.

### Network pharmacology analysis

Drug targets for Epo B were sourced from the SwissTargetPrediction database, utilizing its two-dimensional (2D) structure retrieved from PubChem (PubChem CID: 448013). Targets associated with IBD were identified from the GeneCards database by searching the keyword “Inflammatory Bowel Disease”. Overlapping targets between Epo B and IBD were determined using bioinformatic analyses, and a corresponding Venn diagram was generated. The top 30 common targets were subsequently imported into the STRING database, and the protein category was specified as *Homo sapiens*. The minimum required interaction score was set to “medium confidence (0.4),” and the option to “hide disconnected nodes in the network” was selected to generate the protein–protein interaction (PPI) network visualizing by Cytoscape version 3.9.0. Functional characterization was performed via the DAVID database, implementing comprehensive Gene Ontology (GO) and Kyoto Encyclopedia of Genes and Genomes (KEGG) enrichment studies for the overlapping targets.

### Molecular docking verification

Molecular docking analysis was performed using AutoDock Tools 1.5.6 software.

The three-dimensional (3D) structure of Epo B was retrieved from PubChem. Crystal structures of the target proteins were obtained from the RCSB Protein Data Bank (PDB) database. These receptor structures were refined through the PyMOL software 2.5.0 (Schrödinger, LLC, New York, NY, USA) by removing the original ligands, solvent molecules, and extraneous organic molecules. Subsequent processing of both receptors and ligands was performed using AutoDock Tools, involving hydrogen addition, rotatable bond identification, and docking parameter configuration. Finally, PDBQT files were saved for modeling purposes, and docking scores were calculated to assess the Epo B-target interaction strength. The lowest energy conformation was identified as the most favorable binding model and visualized using PyMOL.

### Thermal shift assay

Recombinant human Caspase-3 protein (15 μg, bs-55032P-Bio, Bioss Inc, Beijing, China) was incubated with either 20 μM Epo B or DMSO for 30 min and heated for 5 min at temperatures ranging from 40 to 67.5 °C in a 96-well Thermal Cycler as described previously.^23^ The reactions were then spun at 13,000 g for 30 min. The supernatant was then analyzed by SDS-PAGE. Thermal stability curves were generated by plotting band intensities against temperature, and the melting temperature (T_m_) was determined from the midpoint of the denaturation profile. A shift in T_m_ relative to the control was interpreted as evidence of direct binding between the protein and the small molecule.

### Statistical analyses

Statistical analyses were performed using GraphPad Prism version 9 (GraphPad Software, Inc.). Data are presented as the mean ± standard deviation (SD) or mean ± standard error of the mean (SEM). All experiments were independently repeated at least three times. Differences between two groups were assessed using two sample *t*-test. Differences among multiple groups were assessed using one-way ANOVA followed by Tukey’s or Dunnett’s test post hoc test, or by two-way ANOVA analysis followed by Sidak’s multiple comparisons test. *P* < 0.05 was considered statistically significant.

## Results

### *In vivo* screening for chemicals that modulating neutrophil homeostasis

**Figure 1:**
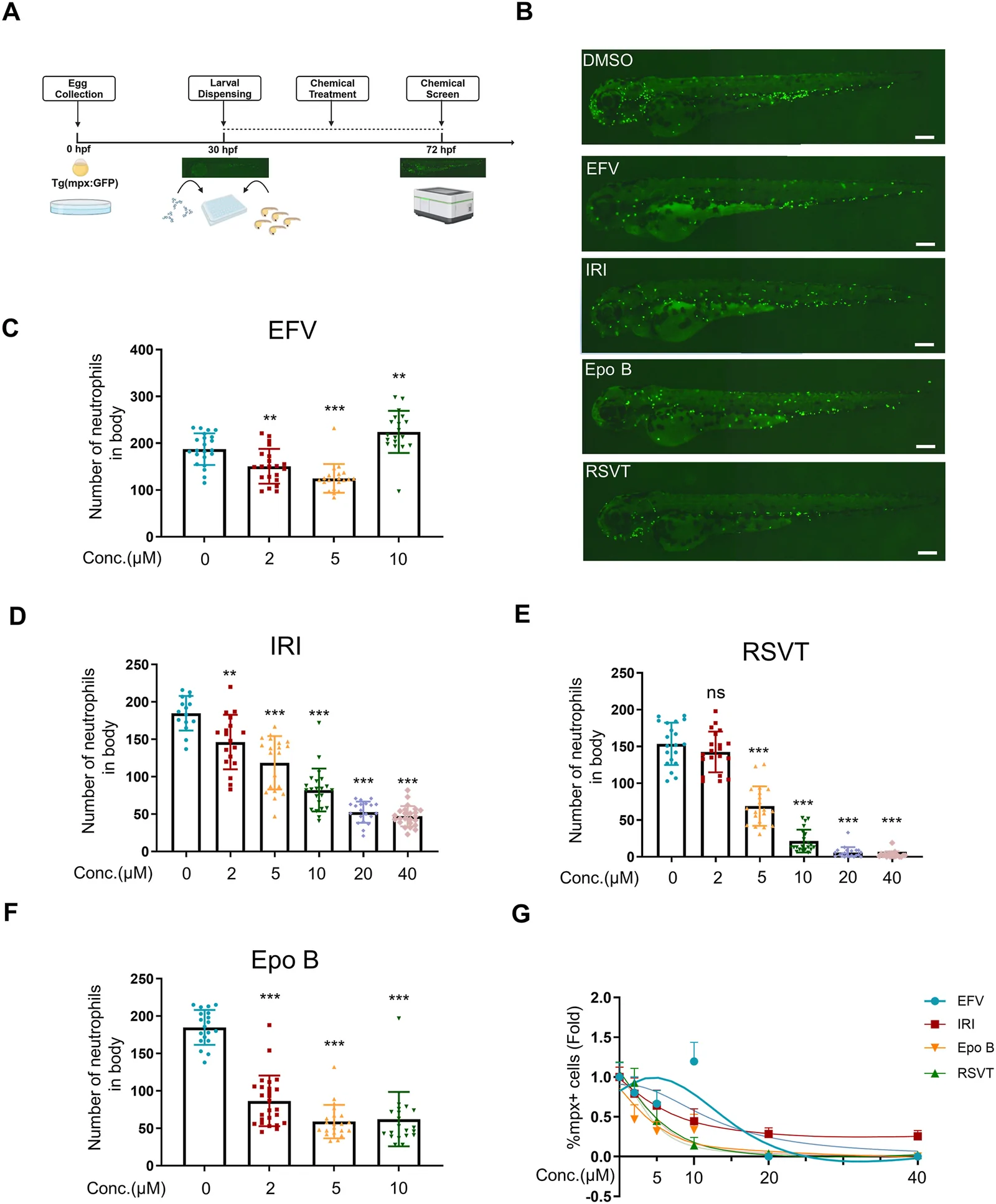
*In vivo* screening for chemicals modulating neutrophil homeostasis. (**A**) Schematic representation of experimental design for high-throughput chemical screening. (**B**) Representative images of *Tg(mpx:GFP)* zebrafish embryos at 3 dpf upon treatment of indicated drugs at 10 μM. Scale bar: 200 μm. (**C–F**) Quantification of total neutrophils in embryos exposed to the specified drugs at indicated concentrations. (**G**) Dose–response analysis of the indicated drugs. Each data point in D–F represents an individual embryo (*n* > 20). Error bars represent mean ± SD. ∗∗*P* < 0.01, ∗∗∗*P* < 0.001; ns, not significant (by one-way ANOVA with Dunnett’s post hoc test compared to DMSO group). Conc, concentration; dpf, days post-fertilization; EFV, efavirenz; Epo B, epothilone B; hpf, hours post-fertilization; IRI, irinotecan hydrochloride; RSVT, Rosuvastatin calcium.

Dysregulation of normal developmental processes frequently influences the onset and progression of pathological changes. Many cellular and molecular mechanisms are shared between homeostasis and disease conditions, which is particularly evident in the extensive crosstalk between neutrophil homeostasis and inflammation.^24^ For instance, granulocyte-colony stimulating factor, which is vital for neutrophil production, differentiation, and activation, significantly promotes inflammatory responses in rheumatoid arthritis.^25^ Additionally, the inhibition of ErbB2 receptor tyrosine kinase increases neutrophil apoptosis and thus reduces inflammation in murine models of acute lung injury.^26^ These findings underscore the potential of identifying the modulators of neutrophilic inflammation by examining their effects on neutrophil homeostasis. Therefore, in this study, a large-scale chemical screening was performed to identify neutrophil homeostasis modulators followed by validation of their effects on the pathogenesis of IBD. We developed a high-throughput chemical screening protocol using the zebrafish neutrophil-specific transgenic line *Tg*(*mpx*:*GFP*)^*i114*^. From an initial screening of 1430 FDA-approved drugs (Fig. 1A) and subsequent manual validation, four chemicals, including Efavirenz (EFV), Irinotecan hydrochloride (IRI), Rosuvastatin calcium (RSVT), and Epo B were found to dose-dependently reduce neutrophil counts (Fig. 1B–F and Table S1). Notably, EFV exhibited biological toxicity at 10 μM, while demonstrating an inhibitory effect on neutrophil numbers at lower, safe dosages (Fig. 1B and C). Finally, a dose-effect analysis based on the same concentration gradient showed clear dose–effect relationships for Epo B, RSVT, and IRI, but no EFV (Fig. 1G).

### Epo B and RSVT mitigate DSS-induced intestinal barrier damage

**Figure 2:**
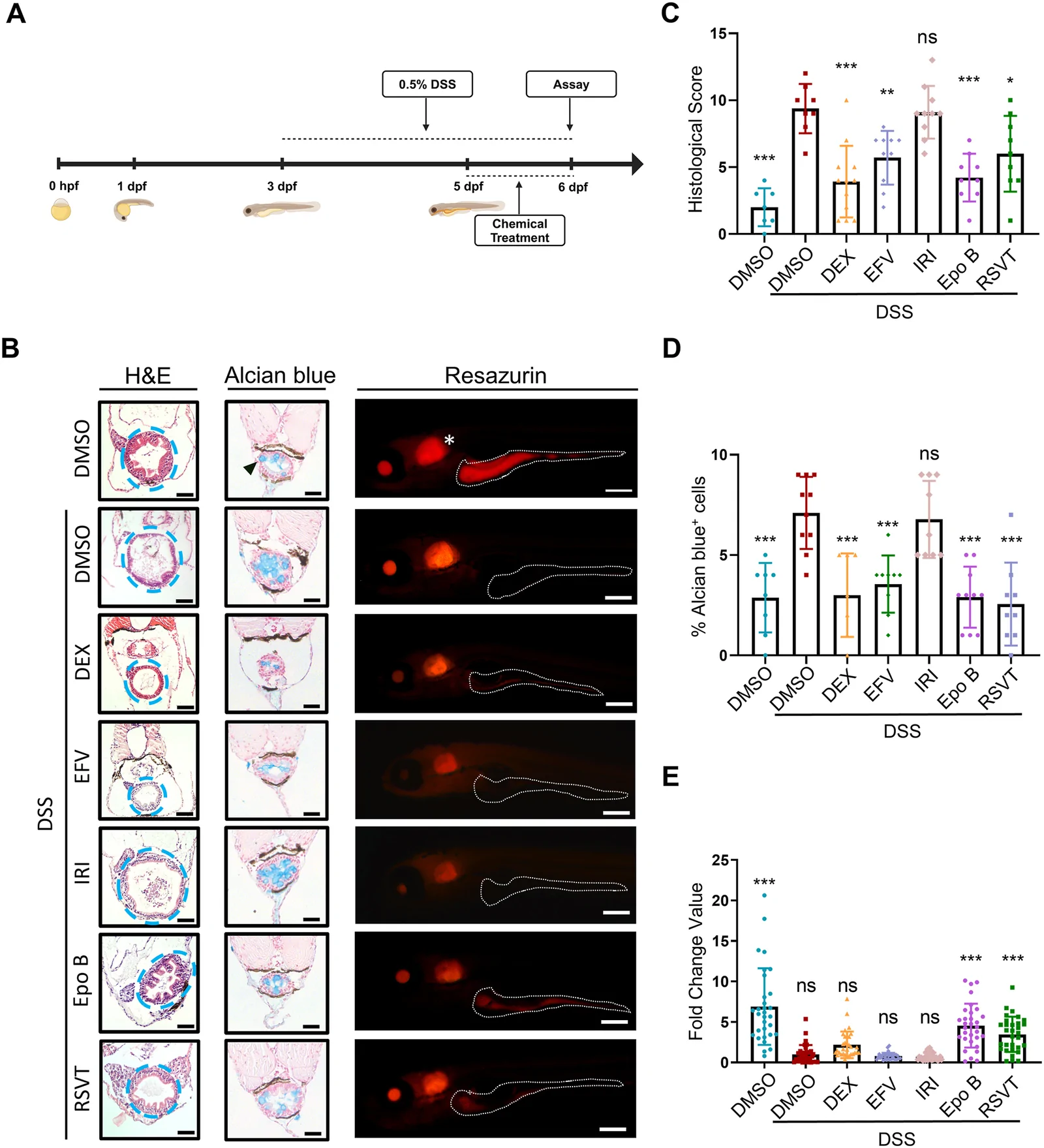
Epo B and RSVT mitigate intestinal barrier damage induced by dextran sodium sulfate (DSS). (**A**) Schematic representation of the experimental design for the DSS-induced zebrafish enterocolitis model and drug treatment. (**B**) Representative images of hematoxylin and eosin (H&E)-stained intestinal bulb sections (left panel), Alcian blue-stained goblet cells in posterior intestine (middle panel), and whole-larvae resazurin staining (right panel) in zebrafish models under indicated treatments. Scale bars: 50 μm (left panel), 25 μm (middle panel), 200 μm (right panel). Dashed blue ovals indicate intestinal lumens. Black arrowhead denotes goblet cells. Dashed white lines and white asterisks denote the intestinal area and otic vesicle, respectively. (**C–E**) Quantification analysis of histological scores (C), Alcian blue-positive cells (D) and resazurin staining (E) for each treatment group outlined in (B). The concentration for each drug was listed in Table S1. Each data point represents an individual larva (*n* ≥ 5). Error bars represent mean ± SD. ∗*P* < 0.05, ∗∗*P* < 0.01, ∗∗∗*P* < 0.001; ns, not significant (by one-way ANOVA with Dunnett’s post hoc test compared to DSS + DMSO group). DEX, dexamethasone.

To investigate the effects of the four identified drugs on IBD, we established a zebrafish larval enterocolitis model by administering 0.5% DSS from 3 to 6 dpf (Fig. 2A). DEX, which ameliorates DSS-induced colonic damage and reduces acute inflammatory responses,^27^ served as a positive control. Histological staining demonstrated that the DSS model group exhibited severe intestinal epithelial damage, as evidenced by a thinner epithelial layer, enlarged intestinal lumen, and an increased number of Alcian blue-stained goblet cells, compared with the control group. Notably, EFV, Epo B, and RSVT, similar to DEX, significantly restored the morphological epithelial injury caused by DSS treatment (left and middle panels of Fig. 2B–D; Fig. S1). Subsequently, the intestinal cell viability was analyzed with a resazurin reduction assay. Strong fluorescent signals were observed in the gut of control larvae, while DSS exposure resulted in a marked reduction of cell viability in the intestine, which was partially but significantly restored by treatment with Epo B or RSVT (right panels of Fig. 2B–E). These findings suggest that Epo B and RSVT can effectively alleviate the pathological damage associated with DSS-induced enterocolitis and promote tissue repair.

### Epo B suppresses the inflammatory response in the zebrafish IBD model

**Figure 3:**
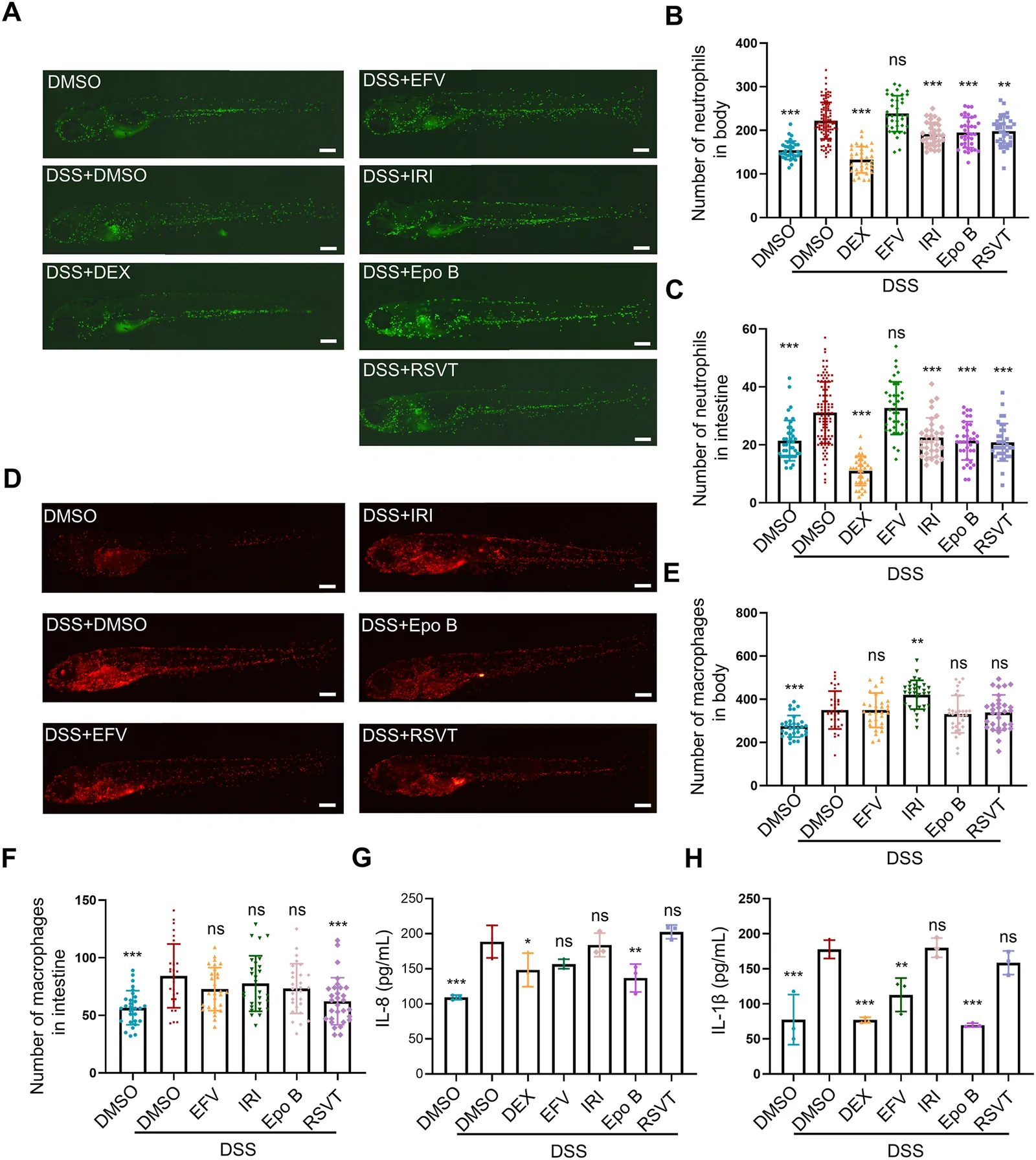
Epo B suppresses the inflammatory response in the zebrafish inflammatory bowel disease (IBD) model. (**A–F**) Representative images (A, D) and quantitative analysis (B, E, whole body; C, F, intestine) of 6-dpf *Tg(mpx:GFP)* or *Tg*(*mpeg1*:*Gal4*);*Tg*(*UAS*:*Nfsb-mCherry*) zebrafish larvae subjected to DSS and/or the treatments of indicated drugs. Scale bar: 200 μm. (**G, H**) Quantitative analysis of IL-8 (G) and IL-1β (H) expression in 6-dpf wild-type zebrafish larvae subjected to the specified drug treatments. Each data point represents an individual larva (B, C, E, F) (*n* > 30) and a biological replicate (G, H) (*n* = 3), respectively. Error bars represent mean ± SD in (B, C, E, F) and SEM in (G, H). ∗*P* < 0.05, ∗∗*P* < 0.01, ∗∗∗*P* < 0.001; ns, not significant (by one-way ANOVA with Dunnett’s post hoc test compared to DSS + DMSO group).

Given that the infiltration of inflammatory leukocytes is a hallmark of the acute phase of IBD, we quantified the neutrophils and macrophages in DSS-treated larvae using *mpx:GFP* and *mpeg1:mCherry* transgenic lines, respectively. Treatment with IRI, Epo B, or RSVT markedly reduced neutrophil abundance in both whole larvae and the intestinal region (Fig. 3A–C). In contrast, intestinal infiltration of macrophage remained unchanged following IRI or Epo B administration but was significantly decreased by RSVT (Fig. 3D–F). To further evaluate drug-induced modulation of intestinal inflammation, we measured the expression of two representative pro-inflammatory cytokines, IL-8 and IL-1β. Notably, Epo B was the only compound that significantly downregulated both cytokines (Fig. 3G and H), exhibiting an anti-inflammatory effect comparable to that of DEX. Collectively, these phenotypic and molecular analyses indicate that Epo B exerts superior efficacy in attenuating IBD-associated inflammation by suppressing neutrophil infiltration and reducing pro-inflammatory cytokine production, thereby supporting its selection for subsequent mechanistic studies.

### Epo B restores gut microbiota defects in DSS-induced IBD

**Figure 4:**
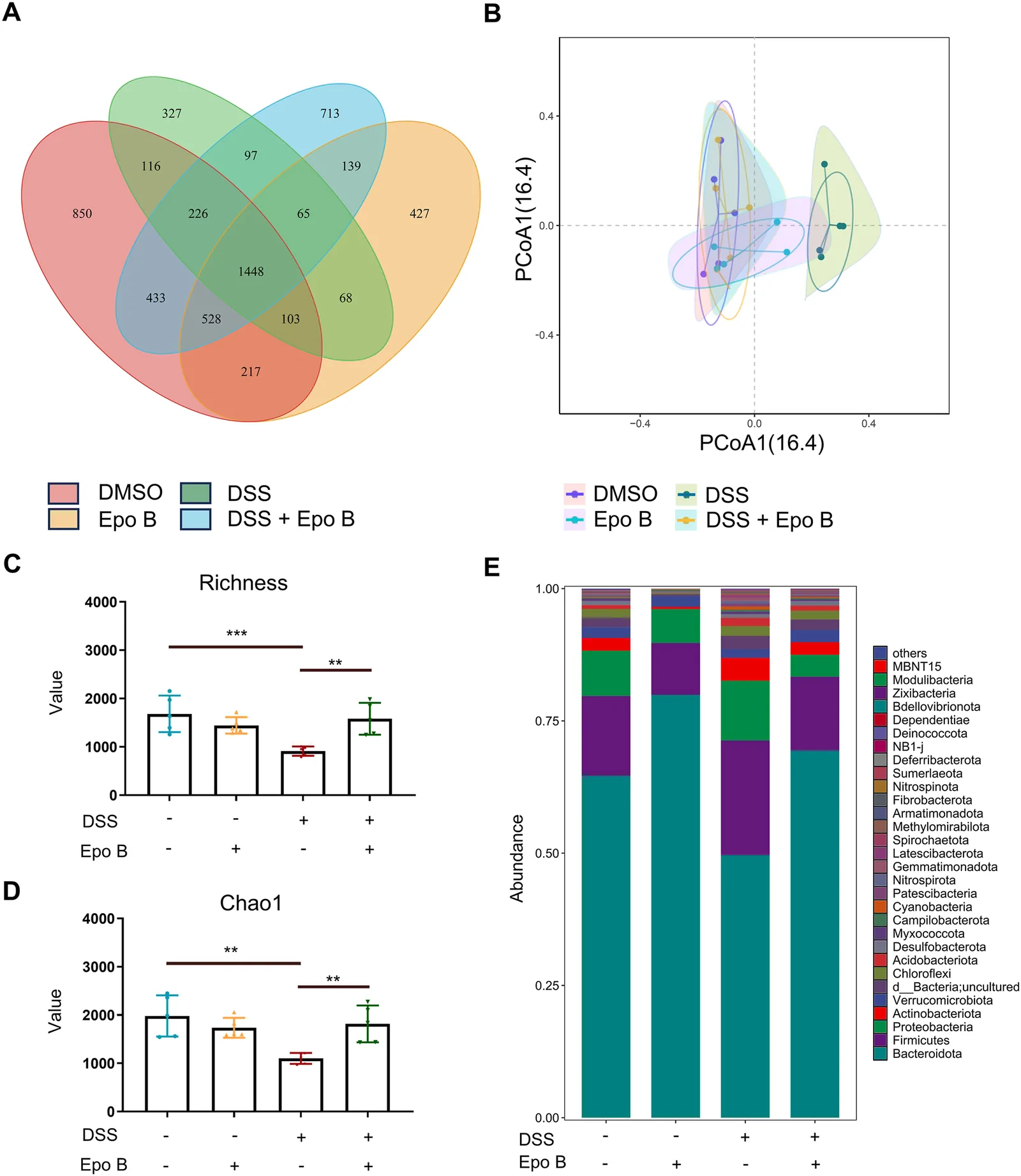
Epo B rectifies gut microbiota defects in dextran sodium sulfate (DSS)-induced zebrafish. (**A**) Venn diagrams of bacterial operational taxonomic units in the gastrointestinal tracts. (**B**) PCoA score plot based on unweighted UniFrac. (**C, D**) Richness index (C) and Chao1 index (D) of α-diversity. (**E**) Phylum-level relative abundance of the gut microbiota. Each data point represents an individual biological replicate (*n* = 5). ∗∗*P* < 0.01, ∗∗∗*P* < 0.001 (by one-way ANOVA with Tukey’s post hoc test).

The imbalance and dysfunction of gut microflora are recognized as key characteristics of DSS-induced colitis.^28^ After confirming intestinal barrier-repairing and immunomodulatory effects of Epo B, we further investigated whether Epo B could remodel the gut microbiota in IBD. We performed 16S rRNA sequencing to analyze the microbiota in the GIT of treated larvae. Exposure to DSS resulted in a significant reduction in the gut microbial diversity, as evidenced by a decrease in the number of OTUs, which was significantly restored by Epo B treatment (Fig. 4A). PCoA using unweighted UniFrac for β-diversity among each genotype revealed that Epo B effectively restored the disrupted gut microbiota induced by DSS to a composition that resembled a normal microbial community (Fig. 4B). Furthermore, Epo B treatment notably restored gut microbial richness and α-diversity, as indicated by α-diversity indices, including the Species Richness index and Chao1 index, which had been significantly reduced in the DSS-induced zebrafish inflammation model (Fig. 4C and D). At the phylum level, the bacterial community detected in all samples (≥ 90%) predominantly consisted of five phyla: Bacteroidota, Firmicutes, Proteobacteria, Actinobacteriota, and Verrucomicrobiota. DSS exposure led to a reduction in the relative abundance of Bacteroidota and an increase in Proteobacteria within the GITs (Fig. 4E). Remarkably, the relative abundances of these bacterial phyla were restored to levels comparable to those in the control group following Epo B treatment. At the order level, GITs in larvae treated with DSS exhibited decreased relative abundance of Bacteroidales and an increased relative abundance of Burkholderiales, both of which were restored by Epo B treatment (Fig. S2). These findings indicate that Epo B effectively mitigates DSS-induced dysbiosis of the gut microbiota in zebrafish colitis.

### Epo B specifically impairs neutrophil homeostasis

The selective effect of Epo B in regulating neutrophil numbers, rather than macrophage numbers, in fish IBD models prompted us to investigate whether this effect arises from its intrinsic ability to modulate neutrophils under homeostatic conditions. We then examined the effect of Epo B on various hematopoietic lineages, including hematopoietic stem/progenitor cells (HSPCs), erythrocytes, thrombocytes, neutrophils, macrophages, and non-hematopoietic endothelial cells, using specific transgenic zebrafish reporter lines. Notably, larvae were treated from 5 to 6 dpf, a treatment condition consistent with that used in the DSS fish model. Treatment with Epo B resulted in a significant decrease in neutrophil numbers, as labeled with *mpx:GFP* (Fig. S3A), while it had no effect on the number of macrophages labeled with *mpeg1:mCherry* (Fig. S3B–S3D), erythrocytes labeled with *gata1:DsRed* (Fig. S3E and S3F), endothelial cells labeled with *kdrl:mCherry* (Fig. S3G and S3H), HSPCs labeled with *cd41: GFP*^low^, or thrombocytes labeled with *cd41:GFP*^high^ (Fig. S3I and S3J). These data suggest that EpoB specifically inhibits neutrophil homeostasis *in vivo*.

To further characterize the impact of Epo B on neutrophil homeostasis, we assessed its effects *in vitro* using purified primary human peripheral neutrophils (Fig. S4), PBMCs, and a panel of hematopoietic and epithelial cell lines. Cell viability was quantified by CCK-8 assays following treatment with graded concentrations of Epo B. Purified human neutrophils displayed markedly higher sensitivity to Epo B, with an IC_50_ of 2.47 nM, compared with the closely related PBMCs and monocytic THP-1 cells (Fig. S5A–S5D). Notably, Epo B exerted minimal effects on the viability of RAW264.7 macrophages, NCM460 colon epithelial cells and 293T cells (Fig. S5E–S5G). These data demonstrate that Epo B selectively reduces neutrophil viability, supporting a cell type–specific regulatory mechanism rather than a broad or nonspecific cytotoxic response.

### Epo B alleviates IBD symptoms in zebrafish by stimulating neutrophil apoptosis

To investigate the cellular and molecular mechanisms underlying Epo B treatment-induced neutrophil reduction phenotypes, network pharmacology was utilized to predict the potential cellular processes and signaling pathways regulated by Epo B in IBD. Using the SwissTargetPrediction database, we predicted 289 potential targets of Epo B, and retrieved 8525 IBD-related genes from the GeneCards database. Subsequently, 238 intersection targets were identified, as illustrated in the Venn diagram presented in Figure S6A. GO and KEGG pathway enrichment analyses of these intersection targets indicated their involvement in multiple cellular processes and signaling pathways, such as the positive regulation of apoptosis, cell differentiation, innate immune response, as well as the phosphoinositide 3-kinase-Akt and mitogen-activated protein kinase signaling pathways (Fig. S6B and S6C).

**Figure 5:**
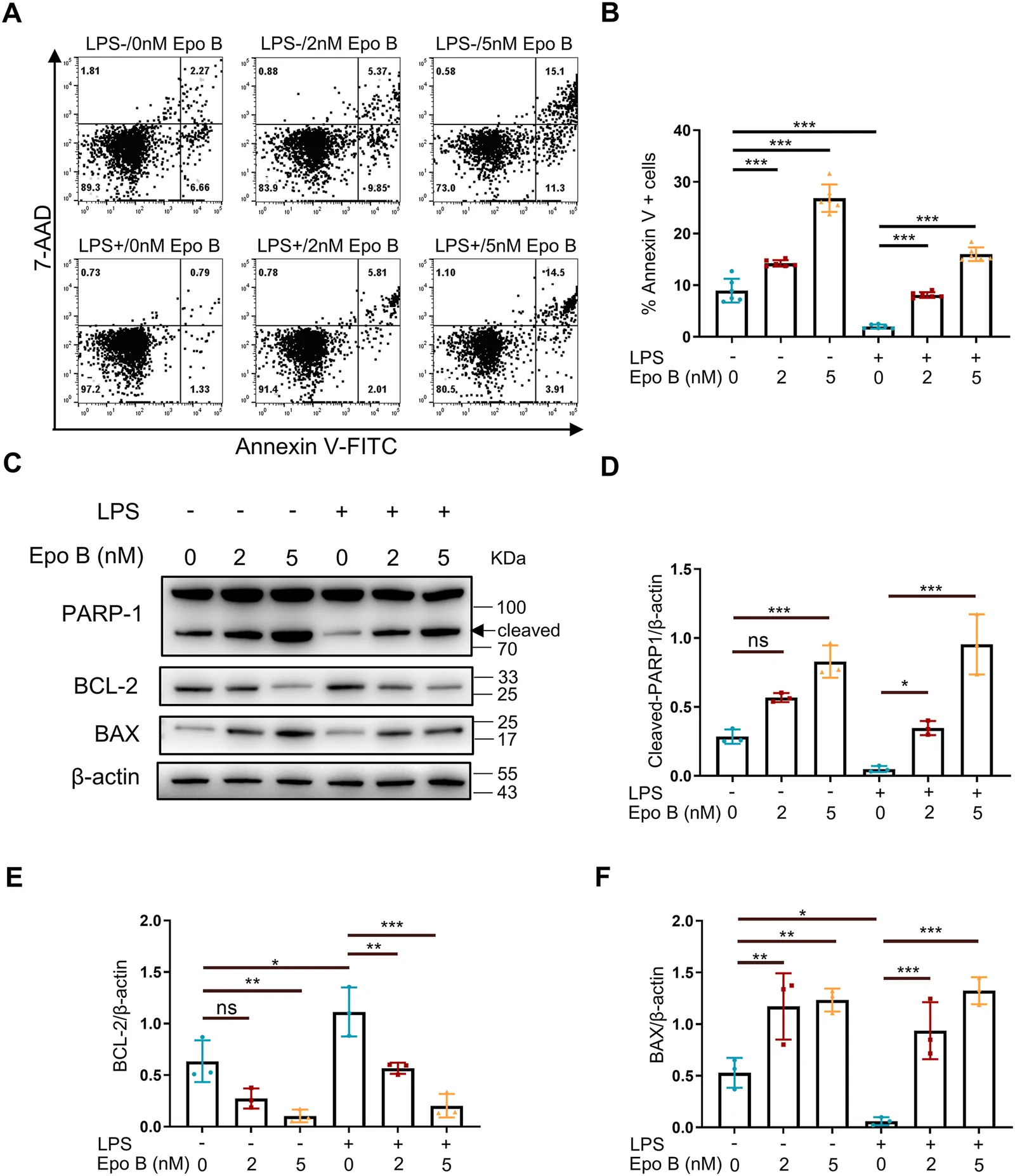
Epo B promotes neutrophil apoptosis *in vitro*. (**A, B**) Representative flow cytometric plots (A) and quantification (B) of Annexin V/7-AAD-stained primary human neutrophils treated with Epo B/LPS. (**C–F**) Representative immunoblots (C) and quantitative analysis of cleaved PARP-1 (D), BCL-2 (E), and BAX (F) in primary human neutrophils treated with Epo B/LPS. Each data point represents an individual biological replicate (*n* ≥ 3). Error bars represent mean ± SD. ∗*P* < 0.05, ∗∗*P* < 0.01, ∗∗∗*P* < 0.001; ns, not significant (by one-way ANOVA with Tukey’s post hoc test). LPS, lipopolysaccharide.

We subsequently examined whether Epo B regulates neutrophil differentiation and cell death, with a particular focus on apoptosis. First, Epo B did not affect neutrophil differentiation in a DMSO-induced differentiation model of HL-60 cells, as evidenced by the expression of CD11b (Fig. S7). Next, we assessed the effect of Epo B on apoptosis in primary human neutrophils under homeostatic and LPS-stimulated conditions, using flow cytometric analysis with 7-AAD/Annexin V staining. Epo B treatment increased the apoptosis rate of human neutrophils in a dose-dependent manner (Fig. 5A and B). No such effect was observed in THP-1, RAW264.7, NCM460, or 293T cells (Fig. S8). These findings strongly indicate that Epo B has a preferential effect on neutrophil apoptosis. To further confirm the effect of Epo B on apoptosis, we analyzed the expression levels of apoptosis-related molecules. Epo B dose-dependently increased the expression of apoptotic markers, including cleaved poly (ADP-ribose) polymerase-1 (PARP1) and Bcl-2-associated X (BAX), while downregulating the expression of the anti-apoptotic protein B-cell lymphoma 2 (BCL-2) (Fig. 5C–F).

**Figure 6:**
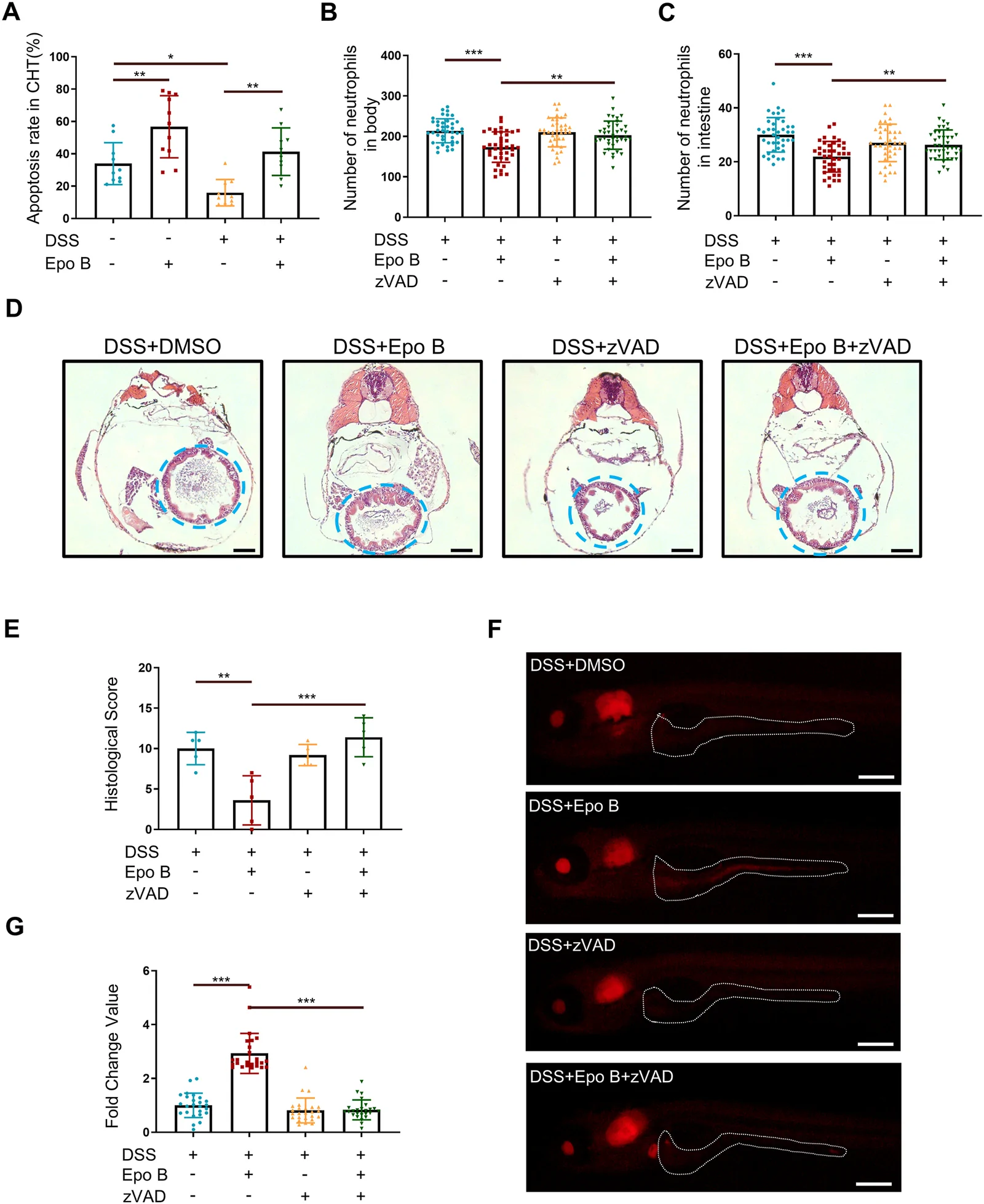
Epo B alleviates inflammatory bowel disease (IBD) symptoms in dextran sodium sulfate (DSS)-induced zebrafish via caspase-dependent neutrophil apoptosis. (**A**) Quantitative analysis of Whole-mount TUNEL staining of 6-dpf *Tg(mpx:GFP)* zebrafish larvae subjected to the DSS/EpoB treatments, with the apoptosis rate defined as the percentage of TUNEL^+^ GFP^+^ neutrophil. (**B, C**) Quantitative analysis (B, whole body; C, intestine) of 6-dpf *Tg(mpx:GFP)* zebrafish larvae subjected to DSS/Epo B/zVAD treatments. **(D)** Representative images of transverse sections from the IB of zebrafish larvae, stained with hematoxylin and eosin. Scale bar: 50 μm. Dashed blue ovals indicate intestinal lumens. **(E)** Quantification of histological scores for each group outlined in (D). **(F, G)** Representative images (F) and quantitative analysis (G) of whole-mount resazurin staining of larvae subjected to DSS/Epo B/zVAD treatments. Scale bar: 200 μm. Dashed white lines denote the intestinal area. Each data point represents an individual larva (*n* ≥ 10). Error bars represent mean ± SD. ∗*P* < 0.05, ∗∗*P* < 0.01, ∗∗∗*P* < 0.001 (by one-way ANOVA with Dunnett’s post hoc test compared to DSS + Epo B group).

Finally, we investigated whether Epo B promotes neutrophil apoptosis in the zebrafish IBD model using whole-mount TUNEL staining. Epo B treatment significantly increased the number of apoptotic mpx^+^ neutrophils compared with untreated larvae (Fig. S9; Fig. 6A). Consistently, co-administration of the pan-caspase inhibitor zVAD markedly reversed the Epo B-induced reduction in neutrophil abundance (Fig. 6B and C) and abolished the enteroprotective effects of Epo B, resulting in aggravated intestinal injury (Fig. 6D–G). Together, these findings demonstrate that Epo B confers intestinal protection predominantly through the induction of neutrophil apoptosis, rather than through effects on neutrophil differentiation or maturation. Moreover, the responsiveness of neutrophils under inflammatory conditions likely reflects an intrinsic, homeostasis-related susceptibility to Epo B-mediated apoptotic regulation.

### Epo B stimulates neutrophil apoptosis by targeting Caspase-3

**Figure 7:**
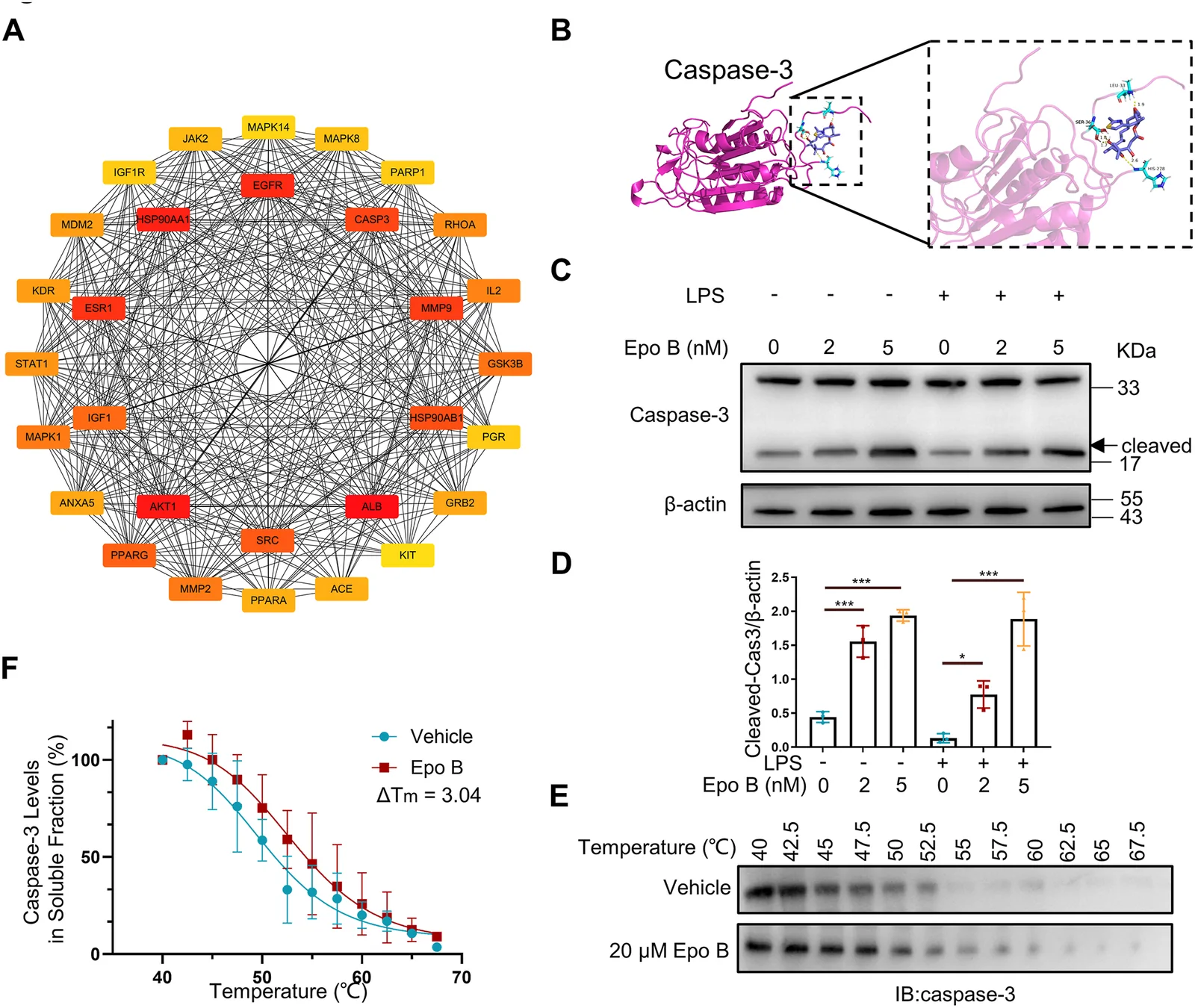
Epo B stimulates neutrophil apoptosis by targeting Caspase-3. (**A**) Protein–protein interaction network of the top 30 hub target genes between Epo B and inflammatory bowel disease (IBD). (**B**) Molecular docking patterns between Epo B and Caspase-3. (**C, D**) Representative immunoblots (C) and quantitative analysis of cleaved Caspase-3 (D) in primary human neutrophils treated with Epo B/LPS. **(E, F)** Representative immunoblots (E) and quantitative analysis (F) of the binding between Epo B and active Caspase-3 assessed by thermal shift assay. Each data point represents an individual biological replicate (*n* = 3). Error bars represent mean ± SD. ∗*P* < 0.05, ∗∗∗*P* < 0.001 (by one-way ANOVA with Tukey’s post hoc test).

To identify the direct target of Epo B in IBD, a PPI network was constructed with 238 intersection targets between Epo B and IBD using the network pharmacology analysis. The top 30 hub genes among the intersection targets were ranked using the degree method in Cytoscape (Fig. 7A and Table S2). Among these genes, CASP3, PPARG and PARP1 are directly associated with the apoptosis regulation. To elucidate the potential interactions between Epo B and these core targets, we conducted a molecular docking analysis with the top 10 hub genes from the PPI. Interestingly, the results revealed the lowest binding energy (−7.06 kcal/mol) between Epo B and Caspase-3, which involves the amino acid residues LEU33, HIS278 and SER36, through hydrogen bonds (Fig. 7B and Table S3). Consistently, the Western blot results further confirmed that Epo B treatment promoted the cleavage of Caspase-3 (Fig. 7C and D). To examine whether Epo B directly interacts with Caspase-3, we performed a thermal shift assay using recombinant Caspase-3 in the presence or absence of Epo B. As shown in Figure 7E and F, the melting temperature (T_m_) of Caspase-3 was approximately 50 °C under control conditions, whereas addition of Epo B increased the T_m_ to 53 °C, indicating enhanced thermal stability of the protein. This upward shift in T_m_ supports direct binding between Epo B and Caspase-3. Together, these data suggest that Epo B stabilizes Caspase-3, potentially facilitating its cleavage and activation, thereby contributing to the pro-apoptotic effect of Epo B on neutrophils during IBD-associated inflammation.

### Epo B ameliorates ulcerative colitis in mice

**Figure 8:**
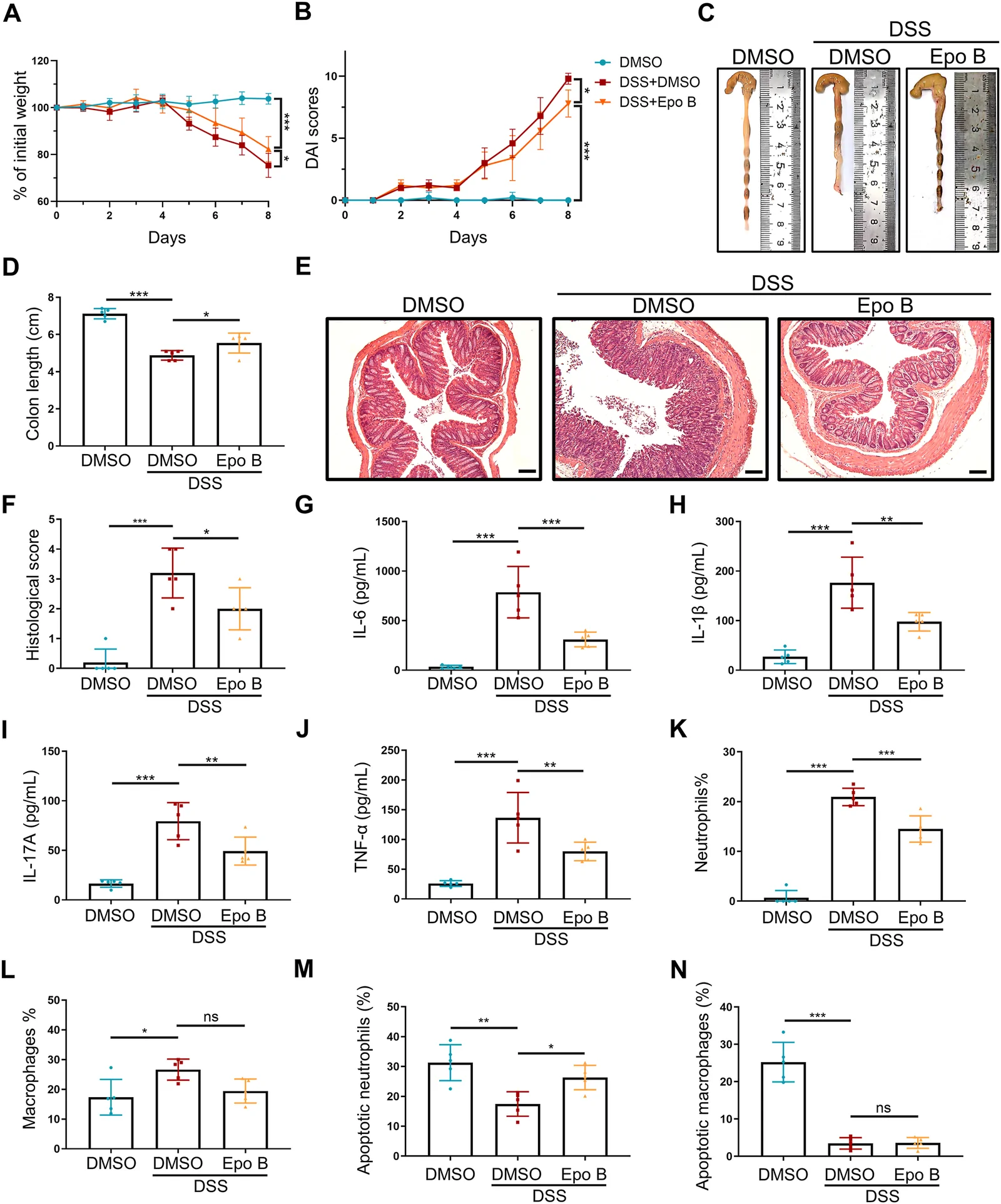
Epo B treatment alleviates dextran sodium sulfate (DSS)-induced ulcerative colitis in mice by specifically stimulating neutrophil apoptosis. **(A, B)** Quantitative analysis body weight changes (A) and disease activity index (DAI) scores (B) of mice during DSS-induced colitis with or without Epo B treatment. **(C, D)** Representative images (C) and length quantitative analysis (D) of colons from each group. **(E, F)** Representative images of transverse sections of colonic tissues (E) and quantification of histological scores (F) from each group. Scale bar: 100 μm. **(G**–**J)** Concentrations of pro-inflammatory cytokines IL-6 (G), IL-1β (H), IL-17A (I), and TNF-α (J) in colonic tissues measured by ELISA. **(K, L)** Representative quantifications of CD45^+^CD11b^+^Ly6G^+^ neutrophils (K) and CD45^+^CD11b^−^F4/80^+^ macrophages (L) in the colon. **(M, N)** Representative quantifications of apoptotic neutrophils (M) and macrophages (N) in the colon. Each data point represents an individual mouse (A, B, D, and F–N). Error bars represent mean ± SD (*n* = 5). ∗*P* < 0.05, ∗∗*P* < 0.01, ∗∗∗*P* < 0.001; ns, not significant (by one-way ANOVA with Dunnett’s post hoc test compared to DSS + DMSO group). DAI, disease activity index.

We further investigated the therapeutic potential of Epo B in mammalian IBD by using a murine model of DSS-induced ulcerative colitis. As expected, mice exposed to DSS exhibited progressive weight loss (Fig. 8A), elevated DAI scores (Fig. 8B), and marked colon shortening (Fig. 8C and D). Histological analysis revealed severe epithelial damage, crypt distortion, and extensive inflammatory infiltration (Fig. 8E and F), all of which were markedly alleviated by Epo B treatment (Fig. 8A–F). Consistently, DSS exposure induced robust upregulation of pro-inflammatory cytokines, including IL-1β, IL-6, IL-17, and TNF-α, whereas Epo B significantly suppressed their expression in colonic tissues (Fig. 8G–J). Flow cytometric analysis showed that Epo B reduced colonic neutrophil accumulation without affecting macrophage proportions (Fig. S10A and S10B; Fig. 8K–L). Moreover, immunofluorescence co-staining demonstrated increased apoptotic neutrophils (Ly6G^+^TUNEL^+^) but not macrophages (F4/80^+^TUNEL^+^) in Epo B-treated mice (Fig. S10C and S10D; Fig. 8M and N). Collectively, these data support our observations in the zebrafish model that Epo B attenuates DSS-induced colitis primarily by promoting neutrophil apoptosis.

## Discussion

In the current study, we conducted a large-scale chemical screen to identify compounds that modulate neutrophil numbers under homeostatic conditions. We further investigated the effects of four drugs identified in this screening using a DSS-induced zebrafish model of IBD. Notably, Epo B exhibited robust therapeutic effects in the zebrafish IBD model, leading to the alleviation of intestinal epithelial barrier disruption, inflammatory response, and rebalancing of the gut microbiota, which was further validated in a DSS-induced ulcerative colitis mouse model. Our findings also show that Epo B preferentially induces neutrophil apoptosis. Here we propose a model for the molecular mechanisms underlying Epo B-regulated IBD pathogenesis (Fig. S11). Specifically, Epo B enhances neutrophil apoptosis, possibly by targeting Caspase-3 and promoting its activation, resulting in a reduction in neutrophil abundance in the intestine and diminishing its role in exacerbating IBD pathogenesis.

Epothilone is a natural 16-membered macrolide compound produced through the metabolism of the cellulose-degrading myxobacterium *Sorangium cellulosum*. Epo B and its derivatives are used as chemotherapeutic agents in various cancers because they interact with and stabilize tubulin, thereby inhibiting cancer cell division.^29^ In the present study, Epo B significantly increased neutrophil apoptosis without affecting terminal differentiation. Given that mature neutrophils do not undergo cell division, the mechanism by which Epo B induces neutrophil death is unlikely to involve cell cycle arrest via tubulin targeting, which results in defective microtubule turnover. Instead, network pharmacology analysis indicated that Epo B may engage apoptosis-related targets, including CASP3 and PPARG. Consistently, molecular docking and thermal shift assays demonstrated that Epo B directly interacts with Caspase-3 and enhances its thermal stability, a mechanism that may facilitate Caspase-3 activation and thereby promote neutrophil apoptosis. Interestingly, Epo B preferentially stimulated neutrophil apoptosis compared to other hematopoietic lineages, as well as non-hematopoietic epithelial and endothelial cells, both *in vivo* in zebrafish larvae (Fig. S3) and *in vitro* in human PBMCs or cell lines (Fig. S5 and S8). The mechanism underlying the selective pro-apoptotic effect of Epo B remains unknown and should be addressed in future studies. We speculate that this bias may arise from the intrinsic differences between neutrophils and other cell types, as mature and aged neutrophils are short-lived and exhibit a high rate of constitutive apoptosis. This predisposition to apoptosis in neutrophils is likely driven by the highly constitutive expression of several pro-apoptotic proteins of the Bcl-2 family, such as Bax and Bak.^30^ These characteristics may render neutrophils more susceptible to Epo B stimulation. Notably, the clinical treatment of cancers with Epo B analogs often results in neutropenia,^31^, ^32^, ^33^ which was considered a myelosuppressive effect common to many chemotherapy drugs, while our findings indicate a direct pro-apoptotic role for Epo B in neutrophils.

Repurposing chemotherapeutic agents at low doses for non-oncological indications is well established in clinical practice.^34^, ^35^, ^36^ Although Epo B has primarily been used as a chemotherapeutic drug for treating cancers, we emphasize that the doses of Epo B used in our study are much lower than its established chemotherapeutic range. We found that intraperitoneal administration of 0.1 mg/kg Epo B was sufficient to significantly alleviate DSS-induced colitis and reduce neutrophil-driven inflammation (Fig. 8) in mice. By contrast, reported efficacious doses of Epo B in mouse tumor models typically fall within the 2.5–20 mg/kg range.^37^, ^38^, ^39^, ^40^ Our study suggests that Epo B exhibits potent anti-inflammatory activity at sub-chemotherapeutic levels. Consistent with this rationale, prior preclinical studies have already explored the therapeutic potential of Epo B in non-oncological conditions at comparable sub-chemotherapeutic doses. For instance, intraperitoneal administration of 0.75 mg/kg and 1.5 mg/kg Epo B improved outcomes in rat spinal cord injury and mouse Parkinson’s disease models, respectively, without lethal or acute toxicity.^41^^,^^42^ Another study reported that 1 mg/kg Epo B significantly ameliorated LPS-induced inflammatory osteolysis in mice, while 5–10 μM was effective *in vitro*, again with no major adverse effects.^43^ Collectively, these findings, together with our own safety observations, provide strong evidence that Epo B can be employed at low, disease-modifying doses across diverse disease contexts without incurring overt toxicity and may potentially be utilized in treating cancer patients also suffering from inflammatory diseases such as IBD.

Our findings demonstrate that Epo B alleviates DSS-induced IBD symptoms by reducing intestinal neutrophil infiltration through the promotion of neutrophil apoptosis. Notably, it has been recognized that neutrophils act as a double-edged sword in IBD, exerting both deleterious and protective effects recently.^2^^,^^3^ On one hand, excessive recruitment and hyperactivation of neutrophils result in the release of ROS, cytotoxic proteases, NETs, and pro-inflammatory cytokines, thereby amplifying inflammation and aggravating mucosal injury. On the other hand, neutrophils contribute to host defense by killing microorganisms and producing pro-resolving mediators that promote intestinal barrier maintenance and tissue repair. The literature also reports conflicting results regarding the consequences of neutrophil depletion. While some studies show that depletion of neutrophils ameliorates DSS-induced colitis in mice or improves disease outcomes in IBD patients,^44^, ^45^, ^46^ others indicate that neutrophil deficiency increases susceptibility to colitis in murine models.^47^, ^48^, ^49^ These discrepancies are likely attributable to differences in disease models and depletion strategies. Importantly, neutrophil heterogeneity provides at least a partial explanation for these dual roles. For example, CD177^+^ neutrophils have been shown to exert protective functions in IBD, characterized by enhanced antibacterial activity, reduced production of IL-6, IL-17A, and IFN-γ, and increased secretion of IL-22, which promotes mucosal repair.^50^^,^^51^ In contrast, low-density granulocytes (LDGs/LDNs) are elevated in IBD patients and are considered to play pro-inflammatory roles in disease pathogenesis.^52^^,^^53^ Considering the complex role of neutrophils in IBD, we speculate that Epo B primarily alleviates colitis by suppressing the deleterious effects of neutrophils, achieved through promoting neutrophil apoptosis and thereby limiting excessive intestinal accumulation, while still preserving protective functions such as bactericidal activity and the facilitation of inflammation resolution. Alternatively, Epo B may selectively target pathogenic neutrophil subsets that exacerbate intestinal inflammation while sparing those with tissue-repairing functions. Future studies will focus on dissecting the impact of Epo B on neutrophil functional duality and subset dynamics.

Neutrophils participate in extensive bidirectional communication with diverse immune populations, including macrophages and T cells, as well as with non-immune stromal populations such as fibroblasts and epithelial cells, thereby influencing IBD pathogenesis.^54^, ^55^, ^56^ Given this cellular complexity, it is plausible that the therapeutic effects of Epo B extend beyond its direct pro-apoptotic action on neutrophils by influencing neutrophil–immune cell crosstalk to exert broader immunomodulatory effects on the inflamed intestinal mucosa. Consistent with this notion, prior studies have shown that Epo B can reshape inflammatory microenvironments in other disease contexts; for example, Epo B modulates microglial activation, depletes bone marrow-derived macrophages, and promotes M1 macrophage polarization in spinal cord injury models.^57^ Additionally, the well-established reciprocal interactions between neutrophils and the gut microbiota in inflammatory disorders,^58^ together with our finding that Epo B restored microbial balance in zebrafish IBD models, suggest an additional layer of regulation. It is conceivable that Epo B may modulate microbially derived metabolites or microbial community structure in ways that reinforce mucosal homeostasis and dampen aberrant inflammation. Although these mechanistic links require rigorous experimental testing, they highlight promising avenues for future investigation.

This study had several limitations. For instance, apart from the induction of neutrophil apoptosis, whether Epo B directly affects neutrophil function to modulate IBD remains unclear and warrants to be addressed in future studies. Furthermore, this study neither precisely identifies the neutrophil subsets targeted by Epo B nor fully excludes the contribution of non-apoptotic mechanisms. These limitations should be addressed through in-depth investigation in future studies.

Despite these limitations, based on chemical screening and subsequent functional and mechanistic studies, we propose that Epo B is a promising therapeutic agent for the treatment of IBD, particularly in the context of co-occurring with cancers.

## Conclusions

This study identified the protective roles and mechanisms of action of Epo B in IBD through high-throughput screening in zebrafish, focusing on its effects on neutrophils. Epo B significantly alleviated the symptoms of DSS-induced colitis by promoting neutrophil apoptosis. These findings provide novel insights and guidance for drug repurposing as well as the development of new therapeutic strategies for IBD.

## CRediT authorship contribution statement

**Zhenting He:** Writing – original draft, Validation, Software, Methodology, Data curation, Conceptualization. **Ziling Deng:** Writing – review & editing, Visualization, Methodology, Data curation, Conceptualization. **Huan Zhang:** Writing – review & editing, Visualization, Data curation, Conceptualization. **Yiming Xiang:** Writing – review & editing, Data curation. **Jiashi Guo:** Writing – review & editing, Data curation. **Yi Chen:** Validation, Software. **Jingjing Qin:** Validation, Software. **Ling Meng:** Validation, Software. **Fengmin Xu:** Validation, Software. **Xiaowen Wang:** Software. **Cheng Zhang:** Resources, Writing – review & editing. **Siyuan Hou:** Validation. **Chengcheng Tao:** Investigation, Validation. **Ruijie Li:** Investigation, Validation. **Jun Chen:** Resources, Writing – review & editing. **Yue Wei:** Resources, Validation. **Wei Wang:** Resources, Validation. **Hao Yuan:** Writing – review & editing, Methodology, Conceptualization. **Chunguang Ren:** Writing – review & editing, Methodology, Conceptualization.

## Ethics declaration

All animal studies were approved by the Institutional Animal Care and Use Committee of Chongqing Medical University (Approval No. IACUG-CQMU-2024-0139). Collection of human peripheral blood sample was approved by the Ethics Committee of The First Affiliated Hospital of Chongqing Medical University (Approval No. K2024-158-01). All donors provided written informed consent prior to specimen acquisition.

## Data availability

The crystal structure of Epo B was retrieved from PubChem (PubChem CID: 448013) and drug targets for Epo B were sourced from the SwissTargetPrediction database. Targets associated with IBD were identified by data deposited in the GeneCards database. The crystal structures of the target proteins were obtained from the RCSB PDB database.

## Funding

This work was supported by 10.13039/501100001809National Natural Science Foundation of China (No. 32170766), 10.13039/501100002865Chongqing Municipal Science and Technology Bureau, China (No. CSTB2025NSCQ-GPX1157), the National Research Center for Translational Medicine at Shanghai, China (No. NRCTM(SH)-2025-08), Open Project of State Key Laboratory of Respiratory Disease, China (No. SKLRD-OP-202602), and 10.13039/501100002858China Postdoctoral Science Foundation (No. 2024MD764043).

## Conflict of interests

The authors declare no competing interests.
