# Supplementary material for: High-throughput chemical screen identifies epothilone B in modulating inflammatory bowel disease by triggering neutrophil apoptosis
Source: Genes Dis. 2025 Dec 23;14(1):102009. doi: 10.1016/j.gendis.2025.102009 (PMC13589075; doi:10.1016/j.gendis.2025.102009)
Supplement: Supplementary file 1 [file mmc1.docx]

**High-Throughput Chemical Screen Identifies Epothilone B in Modulating Inflammatory Bowel Disease by Triggering Neutrophil Apoptosis**

**Supplementary methods**

**Measurement of inflammatory cytokines**

Cytokine levels in zebrafish larvae and mouse colon tissues were determined by Enzyme-Linked Immunosorbent Assay (ELISA). Zebrafish ELISA kits for IL-8 and IL-1β were purchased from Jiangsu Meibiao Biological Technology Company (Jiangsu, China). Mouse ELISA kits IL-6, IL-1β, IL-17 and TNF-α were purchased from Elabscience Biotechnology (Wuhan, China). Briefly, zebrafish larvae collected at 6 dpf were rinsed and homogenized with PBS, and then stored overnight at −20 ℃. Subsequently, the homogenates were centrifuged at 1000×g for 15 min at 4 ℃. Similarly, colon tissues were homogenized in ice-cold lysis buffer and were centrifuged at 12,000×g for 15 min at 4 ℃. The inflammatory cytokine levels in the supernatants were measured according to the manufacturer’s instructions.

**16S rRNA sequencing of intestinal bacteria**

Zebrafish larvae collected at 6 dpf were subjected to microscopy-assisted dissection of the gastrointestinal tracts (GITs), followed by three washes with sterile PBS. The samples were immediately frozen in liquid nitrogen and subsequently stored at -80 °C. Total genomic DNA was extracted using a DNeasy PowerSoil Pro Kit (MoBio Laboratories, Carlsbad, CA, USA), according to the manufacturer’s guidelines. DNA quality was verified by 1% agarose gel electrophoresis, and DNA concentration and purity were determined using a NanoDrop 2000 spectrophotometer (Thermo Fisher Scientific, Waltham, MA, USA). The V3-V4 regions of the 16S rRNA genes were amplified by PCR with primers 341F (5′-CCTAYGGGRBGCASCAG-3′) and 806R (5′-GGACTACHVGGGTWTCTAAT-3′). PCR products were purified, quantified, and homogenized to generate a sequencing library. Quality control of the libraries was performed prior to sequencing using an Illumina NovaSeq 6000 PE250 system. The primary FastQ files were processed and quality-filtered using Trimmomatic, followed by sequence combination as described previously ^1^. Operational taxonomic units (OTUs) were clustered using UPARSE (version 10.0) with a 97% similarity cut-off. Chimeric sequences were identified and deleted using UCHIME (version 8.1). Taxonomic analysis of each 16S rRNA gene sequence was performed by comparison with the Silva 16S rRNA (Release 128) database using the RDP Classifier algorithm (version 2.2), with a confidence threshold of 70%. Bioinformatics analysis was conducted using QIIME, whereas BugBase normalized the OTUs based on the predicted 16S copy number, subsequently predicting the microbial phenotype using the precomputed files. Statistical significance was defined as P < 0.05, with highly significant results defined as P < 0.01. A Venn diagram generated using Mothur was used to identify the core microbiome at the species level. Alpha diversity indices, including richness and Chao1 were calculated with 97% identity using Mothur (version 1.30). Beta diversity analysis was conducted using principal coordinate analysis (PCoA) based on BrayCurtis distances at the relative OTUs level, and unweighted UniFrac distances were used to compare different groups. STAMP v2.1.3 with Welsh’s t-test and one-way ANOVA were used to compare the relative abundances of the predominant bacteria across various groups. P ≤ 0.05 was considered statistically significant.

**Flow cytometry**

Zebrafish larvae collected at 6 dpf were subjected to a 30-min digestion with trypsin after being meticulously chopped up using a sterile scalpel blade. The resulting mixture was then washed with PBS and filtered through a 40 μm nylon mesh to obtain a cell suspension. Fluorescence-activated cell sorting was subsequently performed using the Guava® easyCyte system (Luminex Corporation, Austin, TX, USA). Data analysis was performed using the FlowJo software (TreeStar, Ashland, OR, USA).

**Isolation of human peripheral blood leukocytes and colonic lamina propria immune cells**

Whole blood was collected from healthy donors at The First Afﬁliated Hospital of Chongqing Medical University following the Declaration of Helsinki. Written, informed consent was obtained from each donor prior to the collection of human blood samples, which were de-identified before use. Neutrophils and peripheral blood mononuclear cells (PBMCs) were isolated using a Ficoll-Hypaque gradient, sedimentation with dextran (3% w/v), and hypotonic lysis of erythrocytes. The purity of the isolated neutrophils was determined to be ≥ 90%, as assessed by flow cytometry using anti-human CD11b-APC, CD66b-PE, and CD16-APC antibodies (BioLegend, London, UK). This study was approved by the Ethics Committee of The First Afﬁliated Hospital of Chongqing Medical University (Approval No. K2024-158-01). All donors provided written informed consent prior to specimen acquisition. Colonic lamina propria immune cells were isolated as previously described with minor modifications ^2^. Briefly, the colon was excised, opened longitudinally, and washed with cold PBS to remove fecal residues. After cutting into small fragments, epithelial cells were removed by incubation with 5 mM EDTA (Sigma-Aldrich) and 1 mM DTT (Sigma-Aldrich) in PBS at 37 °C for 20 min. The remaining tissue was digested in RPMI-1640, 0.25 U/mL collagenase D (Sigma-Aldrich), and 0.15 mg/mL DNase I (New England Biolabs, Inc, MA, USA) at 37 °C for 60 min. The cell suspension was filtered through a 70 µm strainer and subjected to 40%/80% Percoll gradient centrifugation. Immune cells collected from the interphase were washed with PBS and assessed by flow cytometry using anti-mouse CD45-FITC, CD11b-APC, Ly6G-PE and F4/80-PerCP/Cy5.5 antibodies (BioLegend)

**Cell culture and treatment**

HL-60 human-derived myeloblastic leukemia cells, THP-1 monocytic cells, HEK embryonic kidney 293T cells, NCM460 normal human colonic epithelial cell line, and RAW264.7 mouse-derived macrophages were obtained from the National Collection of Authenticated Cell	Cultures (Shanghai, China). Cell lines were cultured in RPMI-1640 or Dulbecco’s Modified Eagle’s medium (DMEM, Solarbio) supplemented with 10% fetal bovine serum (FBS) and 1% penicillin/streptomycin at 37°C in a 5% CO2 atmosphere. The cells were regularly tested for mycoplasmas. All the cell lines were used within 10 passages. Neutrophils and PBMCs isolated from human peripheral blood were cultured in RPMI-1640 medium supplemented with 10% autologous serum.

**Cytotoxicity and *in vitro* apoptosis measurement**

For cytotoxicity measurement, cells were seeded in 96‑well plates at a density of 2 ×10^4^ cells per well. Following treatment with Epo B (0-10 nM) for 6 h, the cells were washed with PBS. Subsequently, DMEM or RPMI-1640 medium, along with 10% Cell Counting Kit-8 (CCK-8) reagent (Solarbio), was added to each well according to the manufacturer's instructions. The plates were incubated at 37 ˚C for 4 h, after which the absorbance was measured at 450 nm using a microplate reader (Tecan, Männedorf, Switzerland). To measure cell apoptosis, an Annexin V–FITC apoptosis kit was used according to the manufacturer’s instructions (BioLegend, London, UK). Cells (2 × 10^5^ cells per well) were seeded in six-well plates and treated with Epo B (0, 2, or 5 nM) for 6 h. For human neutrophils, additional exposure groups for lipopolysaccharide (LPS; Sigma-Aldrich) were established, in which LPS (100 ng/ml) was coincubated with the corresponding concentrations of Epo B for 2 h. After treatment, the cells were washed twice with cold PBS, centrifuged at 400×g for 5 min, and resuspended in 1× binding buffer. The cells were stained with 5 μL of Annexin V–FITC and 5 μL of the 7-aminoactinomycin D (7-AAD) reagent at 25℃ for 15 min before flow cytometry analysis.

**Differentiation assay of HL-60 cells**

HL-60 cells (3.5 × 10^5^) were seeded in a T-25 flask and treated with 1.3% DMSO for 5 days to induce differentiation. The cells were also exposed to Epo B (0, 2 and 5 nM) for 24 h (day 4-5). The differentiation of HL-60 cells was assessed by measuring the expression level of CD11b using flow cytometry. Briefly, 1× 10^6^ cells were washed twice with PBS, and stained with anti-CD11b-APC antibody for 30 min at 4 °C in the dark. Unstained cells and cells treated with APC Mouse IgG2b (BioLegend, London, UK) were used as controls. After staining, the cells were resuspended in PBS for flow cytometry analysis. The data were analyzed using the FlowJo software.

**Western blot**

Total proteins from the cell lines were extracted using RIPA lysis buffer containing the protease inhibitor phenylmethanesulfonyl fluoride and phosphatase inhibitors. Protein concentrations were quantified using a Bicinchoninic Acid (BCA) kit (Beyotime, Shanghai, China). Sodium dodecyl sulfate-polyacrylamide gel electrophoresis (SDS-PAGE, 10% gel) was used to resolve proteins, which were transferred onto polyvinylidene fluoride (PVDF) membranes. An Enhanced Chemiluminescence Detection Kit (P0018FM, Beyotime) was used to visualize the protein strips. The primary antibodies used included: anti-PARP1 (A19596, ABclonal, Wuhan, China), anti-Caspase-3 (66470-2-Ig, Proteintech, Wuhan, China), anti-BCL-2 (12789-1-AP, Proteintech), anti-BAX (SZ3-07, HUABIO, Hangzhou, China), and anti-β-actin (20536-1-AP, Proteintech).

**Reference**

1.	Yang Z, Cai T, Li Y, Jiang D, Luo J, Zhou Z. Effects of topical fluoride application on oral microbiota in young children with severe dental caries. *Front Cell Infect Microbiol*. 2023;13:1104343. doi:10.3389/fcimb.2023.1104343

2.	Xu Y, Zheng M. Protocol for isolating innate lymphoid cells from murine lamina propria of the small intestine. *STAR Protoc*. Dec 20 2024;5(4):103360. doi:10.1016/j.xpro.2024.103360

**Supplementary figures**

**Figure S1.** Epo B and RSVT mitigate DSS-induced intestinal pathological injury. (**A-B**) Representative images of transverse sections from the mid-intestine (MI) (A) and posterior intestine (PI) (B) of 6-dpf zebrafish larvae, stained with hematoxylin and eosin. Scale bar, 50 µm. The black arrowhead denotes goblet cells, and the red arrowhead indicates epithelial cells that have shed into the intestinal lumen.

**Figure S2.** Relative abundance of the gut microbiota at the order level in indicated group (n = 5).

**Figure S3.** Epo B impairs neutrophil homeostasis in zebrafish larvae. (**A–B**) Quantification of total fluorescent cells in 6-dpf *Tg(mpx:GFP)* (A) and *Tg*(*mpeg1:Gal4*);*Tg*(*UAS:Nfsb-mCherry*) (B) zebrafish larvae exposed to Epo B by manual counting. (**C–J**) Representative images (C, E, G, I) and quantitative analysis (D, F, H, J) of flow cytometry analysis of 6-dpf *Tg(mpeg1:Gal4);Tg(UAS:Nfsb-mCherry)* (C–D), *Tg*(*gata1:DsRed*) (E–F), *Tg*(*kdrl:mCherry*) (G–H) and *Tg*(*cd41:GFP*) (I–J) zebrafish larvae subjected to the indicated drug treatments. The *cd41:GFP* ^low^ and *cd41:GFP* ^high^ cells represent HSPCs and thrombocytes, respectively. Each data point represents an individual larva (A, B) (n > 30) and a biological replicate (D, F, H, J) (n ≥ 4), respectively. Error bars represent mean ± SD in (A, B) and SEM in (D, F, H, J). **P < 0.01; ns, not significant (by unpaid t-test compared to DMSO group in A, B, D, F and H, by two-way ANOVA with Sidak's multiple comparisons test compared to DMSO group in J). HSPCs, hematopoietic stem/progenitor cells.

**Figure S4.** Flow cytometric Gating strategy for the examination of purity of human primary neutrophils using anti-CD11b, anti-CD66b and anti-CD16. Notably, the purity of neutrophils exceeded 97% when CD66b or CD16 was used as a neutrophil-specific marker.

**Figure S5.** Epo B preferentially suppresses neutrophil cell viability. **(A–G**) Quantitative analysis of cell viability of Epo B-treated primary human neutrophils (A–B), PBMCs (C), THP-1 (D), RAW264.7 (E), NCM460 (F), and 293T cells (G) by CCK-8 assay. Each data point represents an individual biological replicate (n = 3). Error bars represent mean ± SD. *P < 0.05, **P < 0.01, ***P < 0.001; ns, not significant (by one-way ANOVA with Dunnett’s post hoc test compared to DMSO group). PBMCs, peripheral blood mononuclear cells.

**Figure S6.** Network pharmacological analysis of Epo B in IBD. (**A**) Venn diagram of Epo B and IBD targets. (**B–C**) GO function (B) and KEGG pathway (C) enrichment analysis of the overlapping targets between Epo B and IBD.

**Figure S7.** Minimal effect of Epo B on neutrophil differentiation. (**A–B**) Representative images (A) and quantitative analysis (B) of flow cytometry assessing the effects of Epo B on DMSO-induced neutrophil differentiation from HL-60 cells. The differentiation efficiency was quantified based on the ratio of CD11b^+^ cells. Each data point represents an individual biological replicate (n = 3). Error bars represent mean ± SD. **P < 0.01; ***P < 0.001; ns, not significant (by one-way ANOVA with Dunnett’s post hoc test compared to DMSO group).

**Figure S8.** Minimal effects of Epo B on apoptosis in multiple cell lines. (**A–D**) Quantitative analysis of apoptosis, defined as the percentage of Annexin V-FITC-positive cells in THP-1 (A), RAW264.7 (B), NCM460 (C), and 293T cells (D). Each data point represents an individual biological replicate (n = 3). Error bars represent mean ± SD. *P < 0.05 and **P < 0.01; ns, not significant (by one-way ANOVA with Dunnett’s post hoc test compared to DMSO group).

**Figure S9.** Representative images of Whole-mount TUNEL staining of 6-dpf *Tg(mpx:GFP)* zebrafish larvae subjected to the DSS/EpoB treatments. The CHT regions were presented. Scale bar, 100 µm. The white arrowhead denotes the TUNEL and GFP double-positive cells, and the white asterisks denote the non-specific staining signal. CHT, caudal hematopoietic tissue.

**Figure S10.** Epo B induces apoptosis specifically in neutrophils but not macrophages in the colon of DSS-treated mice. **(A–B)** Representative flow cytometric plots of CD45⁺CD11b^+^Ly6G⁺ neutrophils (A) and CD45⁺CD11b^-^F4/80^+^ macrophages (B) in the colon. **(C–D)** Representative images of TUNEL co-stained with Ly6G (C) or F4/80 (D) in colon sections of mice subjected to the DSS/EpoB treatments. Scale bar, 50 µm. The white arrowhead denotes the TUNEL and Ly6G (C) or F4/80 (D) double-positive cells.

**Figure S11.** Diagram of a working model for Epo B-stimulated neutrophil apoptosis to mitigate IBD pathology.

**Supplementary tables**

**Table S1.** Detailed information regarding the dosages and the associated morphological phenotypes of the identified hit compounds

| **Product Name** | **Work Conc.(μM)** | **Neutrophils**  **counts** | **Notes** |
| --- | --- | --- | --- |
| Efavirenz | 2 |  | Significant Deformities ≥ 5 μM |
| Irinotecan HCl Trihydrate | 5 |  | Deformities observed ≥ 10 μM |
| Epothilone B | 2 |  | Significant Deformities ≥ 5 μM; Mortality rate exceeded 1/4 ≥ 10 μM |
| Rosuvastatin Calcium | 5 |  | Hydropericardium ≥ 10 μM; Trunk deformities ≥ 20 μM |

indicate the decreased counts of neutrophils compared with those in control group;

**Table S2.** Target degree values of Top 30 hub genes among the intersection targets between Epo B and IBD

| **Gene Name** | **Degree** | **Gene Name** | **Degree** |
| --- | --- | --- | --- |
| ALB | 254 | RHOA | 120 |
| AKT1 | 234 | STAT1 | 114 |
| HSP90AA1 | 202 | KDR | 108 |
| EGFR | 186 | MDM2 | 108 |
| ESR1 | 180 | ANXA5 | 106 |
| MMP9 | 176 | GRB2 | 106 |
| CASP3 | 172 | PPARA | 100 |
| HSP90AB1 | 168 | ACE | 100 |
| SRC | 164 | JAK2 | 98 |
| PPARG | 162 | MAPK8 | 96 |
| IGF1 | 146 | PARP1 | 94 |
| GSK3B | 134 | PGR | 94 |
| MMP2 | 126 | IGF1R | 94 |
| MAPK1 | 126 | MAPK14 | 92 |
| IL2 | 122 | KIT | 90 |

**Table S3**. Affinities and amino acid sites of ligand-protein interaction in core targets detected by molecular docking

| **Molecule** | **Ligand** | **Binding energy kcal/mol** | **Amino acid binding site** |
| --- | --- | --- | --- |
| Epo B | ALB | -4.6 | LYS413,LYS538 |
|  | AKT1 | -5.9 | LEU52, ASN54 |
|  | HSP90AA1 | -6.12 | ARG201, GLU200, LYS84 |
|  | EGFR | -5.72 | ARG831 |
|  | ESR1 | -5.1 | ARG436 |
|  | MMP9 | -6.37 | HIS236 |
|  | CASP3 | -7.06 | LEU33, HIS278,SER36 |
|  | HSP90AB1 | -5.67 | ARG46 |
|  | SRC | -4.82 | LYS252 |
|  | PPARG | -5.22 | THR297 |
